# Supplementary material for: Genomic profiling in hidradenitis suppurativa: InterOmics pipeline for DNA-RNA sequencing highlights HLA variants, keratin-associated mutations and extracellular matrix alterations as contributing factors to HS pathogenesis
Source: PLoS One. 2025 Jun 20;20(6):e0326458. doi: 10.1371/journal.pone.0326458 (PMC12180620; doi:10.1371/journal.pone.0326458)
Supplement: Attachment — Submitted filename: review.pdf [file pone.0326458.s002.pdf]

I find the goal of this study extremely hard to understand. Is this a paper about (1) a new tool, InterOmics, which happen to be applied to HS as a proof of concept, or about (2) a HS study and its findings, which happen to use InterOmics to obtain the results?

It the answer is (1), what is the tool? Where is the tool? Is there a GitHub repository from which one can install the tool? Is there a tool at all, or is the analysis pipeline the series of bash scripts in the supplementary document? Presumably, this paper was written to encourage other researchers and medical practitioners to use multiomic tools to improve clinical practice – how should they do this?

What are the purposes of the various steps of the algorithm? What is the reasoning behind various choices? The methods section enumerates various steps which were performed, and the supplementary document has some non-commented code that shows how these steps may have been performed in shell. But why these steps and what do they accomplish? Since the tool is presented as a relatively user-friendly toolbox targeted at clinicians, it would be helpful not to assume the reader already knows all the complexities of the process, and to step through the logic.

“Beyond the basic normalization steps, our InterOmics protocol specifically identified genes with a Transcripts Per Million (TPM) expression rate above 10 as a benchmark for significant genetic activity” – what does this mean, precisely? If I understand correctly, the sentence just reports an arbitrary TPM cutoff. Does the protocol (algorithm?) relate to this cutoff choice? This is unclear.

There is an example workflow in document. But at the minimum, it seems natural to have 1. the actual workflow that was used to perform the analysis of the dataset discussed in the paper and 2. an example workflow that shows that the method works on some small synthetic or toy dataset and produces some expected results. How does the MySQL management step play into this? It is mentioned but not characterized.

For articles that characterize tools, some characterization or discussion of advantages over/ comparison to existing tools for analogous purposes is expected. There is certainly a variety of previous tools that combine WES and RNA-seq. It would be appropriate to explain how these tools are different, and what advantages this tool offers.

“The combined use of WES and RNA-seq in clinical approaches increased the number of variants by an average of 20.89%” – would it be possible to characterize the risks of false positives here in more detail? More does not necessarily mean better. Is this good? Is this bad? What performance do other tools achieve? “We enhance confidence by considering only RNAe and ASE variants with a read depth (DP) of 5 or greater in the alternative allele, thus mitigating discrepancies arising from sequencing errors or other potential technical issues” seems like it would account for sequencing errors, but not account for RT or early PCR cycle errors. How would you make the reader confident that an interesting variant is not just the consequence of a single, incidentally highly amplified wrong cDNA? How would you QC for such an issue?

If the answer is (2), then what are the findings? The abstract says “The integration of this data not only advances our comprehension of HS's underlying mechanisms but also demonstrates the potential of

multiomics to improve personalized medical approaches." The conclusions say "The capability of InterOmics to integrate multiomic data has the great potential to elucidate the pathogenesis of complex diseases, such as HS, from an omic standpoint. Therefore, for conditions like HS, which involve intricate molecular and environmental interactions, such a comprehensive analysis is important." In what way does the paper accomplish this? I understand that the procedure characterizes a diversity of variants. But the authors do not take the final step and connect show that the some or all of these results are sensible in explaining the pathogenesis of HS.

In other words: presenting the tool also requires explaining how a user should interpret its results, and I do not think this last essential connection to the disease is really explained; the paper jumps from presenting tables of variants – which can reasonably be called "personalized genomic/transcriptomic profiles" – to saying the tool "offer[s] deep insights into the unique genetic landscapes of these individuals." I am not clear on what these insights are, or how one should obtain such insights from applying the workflow to their own data.

Where are the data? New patient data were collected for the study, but this is not discussed in the manuscript or submission disclosures, which state "all data are fully available without restriction." I realize that releasing human patient data involves various challenges. But the reader should be able to obtain the data the conclusions are based on, or at the very minimum understand what they would need to do to obtain the data.

Could the authors comment on what seems to be a contradiction between using GRCh38 to quantify RNA but also exploring complex differences between the patient genome and GRCh38. I would intuitively think that if the pipeline is generating an individual genomic profile, the quantification would be with reference to that profile, instead of something else. Does it make a difference?

Some broken references e.g. line 260, 283. Various proofreading issues e.g. line 266. "InterOMICs" or "InterOmics"? If "GRChg38" is the name of a genome, it is unexpected and should be explained/cited. WSX = unexplained abbreviation? TBS = typo of TFBS?

Some oddities in table S2 findings. nhr-6, a C. elegans gene? AT5G05790, an Arabidopsis gene? YKL222C, a yeast gene? Maybe there is some homology argument, but it is not really clear how his should be interpreted in terms of human genetics, and the authors do not explain.

TF2DNA is a bare link to an inaccessible website. Was this database published somewhere? Could other databases be used?

Various other bare links. (The TCAG one may even be blocked by modern browsers because it is a download from a non-secure http website). Is this zip file versioned? Is it identical to the file linked in the 2018 paper? Have there been updates in the last six years? When was it accessed for this paper's analysis? Will the file be available and identical in the future? Etc. Various other bare links, databases, R packages discussed without version information.

Fig 3 is very hard to understand without carefully reading the caption. I expect that there is a better way to illustrate these distinctions – perhaps by showing the sort of data these variants can generate? Or distinguishing the different chromosomes? As it reads, it conflates technology and biology (whether some variant is *not present* or merely *not observed* because of a lack of reads) and the distinction between e.g. DNA-only and [rare recovery] RNA editing is unclear.

This is partially a stylistic point but I think crucial to the paper's scope: there is a tremendous amount of superlative language. “innovative”, “promises to uncover previously unrecognized molecular signatures”, “profound insight”, “evidence to the potential… in advancing personalized medicine”, “delve deep”, “paving the way for novel insights and novel potential therapeutic strategies”, “a transformative advancement”, “pivotal, not merely for its technical value but for its profound impact on expanding our genomic insight”, “paradigm shift”, “its contributions to the field of genomics are expected to grow, marking a promising era of discovery and innovation”, “designed to revolutionize” and so on. There are all specific claims and conclusions about the method's impact and utility.

The review is not based on impact. But it is based on making sure that the claims in the paper are supported, e.g.:

- *What are the main claims of the paper and how significant are they for the discipline?*
- *Are the claims properly placed in the context of the previous literature? Have the authors treated the literature fairly?*
- *Do the data and analyses fully support the claims? If not, what other evidence is required?*

If the authors repeatedly claim broad significance and revolutionary novelty, then please provide evidence, which must necessarily address superiority over the rest of the field and concrete impact im advancing personalized medicine. I do not really see this in the current version.
