# Supplementary material for: Genomic profiling in hidradenitis suppurativa: InterOmics pipeline for DNA-RNA sequencing highlights HLA variants, keratin-associated mutations and extracellular matrix alterations as contributing factors to HS pathogenesis
Source: PLoS One. 2025 Jun 20;20(6):e0326458. doi: 10.1371/journal.pone.0326458 (PMC12180620; doi:10.1371/journal.pone.0326458)
Supplement: Attachment — Submitted filename: R1.pdf [file pone.0326458.s004.pdf]

Point-to-point response to Reviewers

Reviewer#1

Comment

I find the goal of this study extremely hard to understand. Is this a paper about (1) a new tool, InterOmics, which happen to be applied to HS as a proof of concept, or about (2) a HS study and its findings, which happen to use InterOmics to obtain the results?

It the answer is (1), what is the tool? Where is the tool? Is there a GitHub repository from which one can install the tool? Is there a tool at all, or is the analysis pipeline the series of bash scripts in the supplementary document? Presumably, this paper was written to encourage other researchers and medical practitioners to use multiomic tools to improve clinical practice – how should they do this?

What are the purposes of the various steps of the algorithm? What is the reasoning behind various choices? The methods section enumerates various steps which were performed, and the supplementary document has some non-commented code that shows how these steps may have been performed in shell. But why these steps and what do they accomplish? Since the tool is presented as a relatively user-friendly toolbox targeted at clinicians, it would be helpful not to assume the reader already knows all the complexities of the process, and to step through the logic.

“Beyond the basic normalization steps, our InterOmics protocol specifically identified genes with a Transcripts Per Million (TPM) expression rate above 10 as a benchmark for significant genetic activity” – what does this mean, precisely? If I understand correctly, the sentence just reports an arbitrary TPM cutoff. Does the protocol (algorithm?) relate to this cutoff choice? This is unclear.

There is an example workflow in document. But at the minimum, it seems natural to have 1. the actual workflow that was used to perform the analysis of the dataset discussed in the paper and 2. an example workflow that shows that the method works on some small synthetic or toy dataset and produces some expected results. How does the MySQL management step play into this? It is mentioned but not characterized.

For articles that characterize tools, some characterization or discussion of advantages over/ comparison to existing tools for analogous purposes is expected. There is certainly a variety of previous tools that combine WES and RNA-seq. It would be appropriate to explain how these tools are different, and what advantages this tool offers.

“The combined use of WES and RNA-seq in clinical approaches increased the number of variants by an average of 20.89%” – would it be possible to characterize the risks of false positives here in more detail? More does not necessarily mean better. Is this good? Is this bad? What performance do other tools achieve? “We enhance confidence by considering only RNAe and ASE variants with a read depth (DP) of 5 or greater in the alternative allele, thus mitigating discrepancies arising from sequencing errors or other potential technical issues” seems like it would account for sequencing errors, but not account for RT or early PCR cycle errors. How would you make the reader confident that an interesting variant is not just the consequence of a single, incidentally highly amplified wrong cDNA? How would you QC for such an issue?

If the answer is (2), then what are the findings? The abstract says “The integration of this data not only advances our comprehension of HS's underlying mechanisms but also demonstrates the potential of multiomics to improve personalized medical approaches.” The conclusions say “The capability of InterOmics

to integrate multiomic data has the great potential to elucidate the pathogenesis of complex diseases, such as HS, from an omic standpoint. Therefore, for conditions like HS, which involve intricate molecular and environmental interactions, such a comprehensive analysis is important." In what way does the paper accomplish this? I understand that the procedure characterizes a diversity of variants. But the authors do not take the final step and connect show that the some or all of these results are sensible in explaining the pathogenesis of HS.

In other words: presenting the tool also requires explaining how a user should interpret its results, and I do not think this last essential connection to the disease is really explained; the paper jumps from presenting tables of variants – which can reasonably be called "personalized genomic/transcriptomic profiles" – to saying the tool "offer[s] deep insights into the unique genetic landscapes of these individuals." I am not clear on what these insights are, or how one should obtain such insights from applying the workflow to their own data.

Response

We appreciate the Reviewer's insightful feedback, which helps clarify the primary objective of our study. Our study primarily focuses on integrating whole exome sequencing (WES) and RNA sequencing (RNA-seq) to better understand the genetic basis of Hidradenitis Suppurativa (HS), a model of chronic autoinflammatory skin condition. The InterOmics pipeline is presented as a structured bioinformatics framework rather than a standalone software tool. It serves as a methodological approach to integrating genomic and transcriptomic data, improving variant classification and interpretation within complex diseases like HS. While we introduce InterOmics as a robust tool for analysis, the primary goal of the study is to apply this approach to uncover novel genetic and transcriptomic alterations in HS.

Comment

Where are the data? New patient data were collected for the study, but this is not discussed in the manuscript or submission disclosures, which state "all data are fully available without restriction." I realize that releasing human patient data involves various challenges. But the reader should be able to obtain the data the conclusions are based on, or at the very minimum understand what they would need to do to obtain the data.

Response

Our WES and RNA-seq data contain sensitive human genomic information and cannot be publicly shared due to ethical restrictions. However, data access can be granted through a controlled request process. Researchers can request access by contacting the corresponding author and providing:

1. A brief research proposal outlining intended use.

2. Institutional ethical approval for human genomic research.

3. Agreement to comply with patient privacy regulations.

Processed results, summary statistics, and multiomic classifications are fully available within the manuscript and supplementary materials, ensuring transparency while maintaining ethical integrity.

Comment

Could the authors comment on what seems to be a contradiction between using GRCh38 to quantify RNA but also exploring complex differences between the patient genome and GRCh38. I would intuitively think that if the pipeline is generating an individual genomic profile, the quantification would be with reference to that profile, instead of something else. Does it make a difference?

Response

Using GRCh38 as a reference genome for both WES and RNA-seq ensures consistency in variant calling and transcript quantification. A patient-specific genome would introduce alignment biases, impacting:

1. Isoform detection and transcriptome completeness.

2. Reproducibility across datasets.

This approach reduces false positives, allowing reliable RNA-specific variant detection (e.g., Allele-specific expression -ASE- and RNA editing -RNAe-) while maintaining alignment accuracy.

Comment

Some broken references e.g. line 260, 283. Various proofreading issues e.g. line 266. “InterOMICs” or “InterOmics”?

Response

We apologize for that. The name of the pipeline was revised accordingly throughout the text and fixed the broken references.

Comment

If “GRChg38” is the name of a genome, it is unexpected and should be explained/cited. WSX = unexplained abbreviation? TBS = typo of TFBS?

Response

We apologize, we corrected and clarified this in the text accordingly.

- Genome Reference Consortium Human Build 38 (GRChg38)

Comment

Some oddities in table S2 findings. nhr-6, a C. elegans gene? AT5G05790, an Arabidopsis gene? YKL222C, a yeast gene? Maybe there is some homology argument, but it is not really clear how his should be interpreted in terms of human genetics, and the authors do not explain.

Response

The Reviewer pointed out several unexpected gene identifiers in Table S2, such as nhr-6 (a Caenorhabditis elegans gene), AT5G05790 (an Arabidopsis thaliana gene), and YKL222C (a Saccharomyces cerevisiae gene). Upon reviewing our analysis, we found that these occurrences resulted from the way motifbreakR, the tool we used for predicting TFBS disruptions, generates its output. If the species filter is not explicitly applied, motifbreakR may return motif matches based on homology to known transcription factor motifs across multiple species, rather than restricting predictions to human TFBS disruptions alone.

Upon conducting a more detailed review of our TFBS analysis, we identified additional findings that raised concerns about the accuracy and interpretability of these results. Specifically, we found that:

1. The inclusion of non-human gene motifs created inconsistencies that complicated interpretation in a human disease context.

2. The predictive nature of TFBS disruption analysis led to potential overpredictions, increasing the risk of false-positive results.

3. Some motifbreakR findings lacked robust validation within known human regulatory networks, making their biological significance uncertain.

Given these concerns, we have made the decision to remove the TFBS analysis from our pipeline to maintain the scientific integrity of our study. While transcription factor binding site disruptions remain an important topic in multiomic variant analysis, we believe that our current dataset and methodological framework do not yet provide sufficiently reliable TFBS predictions that would allow for meaningful interpretation in the context of HS pathogenesis. Rather than presenting findings that may introduce ambiguity, we opted for a more conservative approach that prioritizes high-confidence variant classifications within the scope of our study.

To ensure that this is reflected in the manuscript, we have made the following revisions:

• The TFBS analysis has been removed from our pipeline and from Supplementary Table S2.

• The methods and results sections have been updated accordingly.

• All findings now strictly pertain to human-relevant genomic and transcriptomic data to avoid potential misinterpretations.

Comment

TF2DNA is a bare link to an inaccessible website. Was this database published somewhere? Could other databases be used?

Various other bare links. (The TCAG one may even be blocked by modern browsers because it is a download from a non-secure http website). Is this zip file versioned? Is it identical to the file linked in the 2018 paper? Have there been updates in the last six years? When was it accessed for this paper's analysis? Will the file be available and identical in the future? Etc.

Response

The long-term availability and versioning of third-party resources are indeed critical for ensuring the reproducibility of research findings. Below, we clarify the status of TF2DNA, as well as other linked resources, and how we have addressed these concerns in the manuscript. During our review, we confirmed that TF2DNA, which was initially cited via a direct URL, is no longer accessible through the provided link. Upon further investigation, we located the original publication describing this database and updated our manuscript to cite the peer-reviewed article associated with TF2DNA, rather than relying on a direct web link. Unfortunately, the alternative URL referenced in the paper is also no longer functional, suggesting that this resource is no longer actively maintained. Regarding other external resources used in our study, we acknowledge that we cannot guarantee their continued availability, particularly in the exact versions we used. Some datasets, such as the one hosted on The Centre for Applied Genomics (TCAG) website, are accessed via direct downloads from non-secure HTTP links, which modern browsers may restrict or block. In response to the Reviewer’s concerns, we have taken the following steps to improve clarity and reproducibility:

1. Versioning and Timestamping

o We have included explicit information about the version of each external dataset used in our analysis and the date it was accessed.

o This ensures that future researchers can verify whether updates have occurred since our study was conducted.

2. Availability of Key Resources

o Where possible, we have provided citations for databases that have associated publications, rather than relying solely on web links that may become obsolete.

3. Reproducibility Considerations

o While we cannot guarantee long-term access to third-party datasets, we have ensured that all critical input files necessary for reproducing our analysis are stored within our institutional data repository.

o Researchers interested in using the same dataset versions can contact the corresponding author to obtain a snapshot of the files used at the time of our study.

4. Correction of Bare Links

o We have replaced bare URLs with proper citations where applicable, ensuring that references remain valid even if web pages become inactive.

We acknowledge the importance of database stability and accessibility in research, and while we have taken steps to ensure reproducibility, we also recognize the limitations imposed by external resources that are beyond our control. The revisions made to our manuscript now provide a more transparent discussion of dataset versioning, availability, and how users can access similar data in the future.

Comment

Various other bare links, databases, R packages discussed without version information.

Response

We corrected accordingly in the text of the manuscript

Comment

Fig 3 is very hard to understand without carefully reading the caption. I expect that there is a better way to illustrate these distinctions – perhaps by showing the sort of data these variants can generate? Or distinguishing the different chromosomes? As it reads, it conflates technology and biology (whether some variant is not present or merely not observed because of a lack of reads) and the distinction between e.g. DNA-only and [rare recovery] RNA editing is unclear.

Response

Dear Reviewer, we have made many changes to the manuscript, and as a result Figure3 has been removed.

Comment

This is partially a stylistic point but I think crucial to the paper's scope: there is a tremendous amount of superlative language. "innovative", "promises to uncover previously unrecognized molecular signatures", "profound insight", "evidence to the potential… in advancing personalized medicine", "delve deep", "paving the way for novel insights and novel potential therapeutic strategies", "a transformative advancement", "pivotal, not merely for its technical value but for its profound impact on expanding our genomic insight", "paradigm shift", "its contributions to the field of genomics are expected to grow, marking a promising era of discovery and innovation", "designed to revolutionize" and so on. There are all specific claims and conclusions about the method's impact and utility.

The review is not based on impact. But it is based on making sure that the claims in the paper are supported, e.g.:

• What are the main claims of the paper and how significant are they for the discipline?

• Are the claims properly placed in the context of the previous literature? Have the authors treated the literature fairly?

• Do the data and analyses fully support the claims? If not, what other evidence is required?

If the authors repeatedly claim broad significance and revolutionary novelty, then please provide evidence, which must necessarily address superiority over the rest of the field and concrete impact im advancing personalized medicine. I do not really see this in the current version.

Response

We sincerely appreciate the Reviewer's careful critique regarding the use of superlative language in our manuscript. We recognize that strong claims about the novelty, impact, and transformative nature of our approach must be fully supported by evidence, and we agree that unsubstantiated claims should be avoided to maintain credibility.

Upon reviewing our manuscript in light of this feedback, we acknowledge that certain phrases—such as "transformative advancement," "pivotal," "paradigm shift," and "revolutionize"—may give an impression of overstatement that is not explicitly justified through comparative analyses or benchmarking against existing methodologies. While we firmly believe that our approach contributes meaningfully to the field of

genomics and personalized medicine, we have taken a more measured approach in our revised manuscript to ensure that our claims are aligned with the strength of our findings and the broader context of the literature.

1. Clarifying the Main Claims and Their Significance

Our study demonstrates the added value of integrating whole exome sequencing (WES) and RNA sequencing (RNA-seq) data to enhance variant detection, classification, and functional interpretation in the context of hidradenitis suppurativa (HS). Specifically, we show that:

• The integration of WES and RNA-seq increases the detection of potentially relevant genetic variants by an average of 20.89% compared to WES alone.

• Allele-specific expression (ASE) and RNA editing (RNAe) events provide additional layers of molecular insight that are not captured through traditional DNA-based analyses.

• Our categorization system for genomic and transcriptomic variants facilitates the identification of disease-relevant regulatory mechanisms, particularly in post-transcriptional gene regulation.

While these findings are significant within the domain of multiomic variant analysis, we acknowledge that they do not constitute a fundamental paradigm shift in genomics or revolutionize personalized medicine. Instead, they represent an incremental yet meaningful improvement in how multiomic data can be used to study complex diseases.

2. Ensuring Fair Representation of Previous Literature

We also appreciate the Reviewer's concern regarding the placement of our work within the broader context of existing literature. Our approach builds upon previous studies that have integrated WES and RNA-seq in disease research, and we recognize that similar methodologies have been employed in other genetic and transcriptomic studies of inflammatory diseases.

3. Strengthening the Evidence Base for Impact Claims

While our findings support the utility of multiomic integration, we recognize that our study does not directly demonstrate clinical impact or superiority over existing tools.

To ensure appropriate framing, we have revised our manuscript to:

• Clearly distinguish between findings supported by data and potential future applications.

• Avoid speculative or exaggerated claims about the immediate impact of our study.

• Emphasize that while our method enhances genetic variant interpretation, further validation is needed to translate these findings into clinical practice.

4. Revisions to Improve Precision and Objectivity

To address the Reviewer's concerns, we have systematically removed or rephrased superlative language that may have overstated our contributions. Specifically, we have:

• Replaced phrases like "paradigm shift" with a more measured discussion of our contributions.

• Removed excessive references to transformation, revolution, or groundbreaking impact unless directly supported by comparative analysis.

• Ensured that all claims are supported by data, literature, or explicitly framed as future research directions.
