# Supplementary material for: Genomic profiling in hidradenitis suppurativa: InterOmics pipeline for DNA-RNA sequencing highlights HLA variants, keratin-associated mutations and extracellular matrix alterations as contributing factors to HS pathogenesis
Source: PLoS One. 2025 Jun 20;20(6):e0326458. doi: 10.1371/journal.pone.0326458 (PMC12180620; doi:10.1371/journal.pone.0326458)
Supplement: Attachment — Submitted filename: Response_to_Reviewers_auresp_2.docx [file pone.0326458.s006.docx]

**Reply point-by-point**

**Reviewer #1**

**Comment**

I want to begin by saying that I think the authors did a superb job with this revision. The manuscript is now terse, clear, and focused. The improvement is considerable, and I am mostly satisfied with the changes made to address my concerns.

**Reply**

Dear Reviewer, thank you very much for your appreciation of our revision.

**Comment**

Still a few minor errors:

154 ", which consists on variants that it homozygous "

"GRCh38" not "GRChg38"

**Reply**

We corrected the mistake accordingly

**Comment**

I think there are two remaining basic issues. The first is crucial to me, on the second one I will defer to the editor.

This paper is about a particular structured framework for thinking about DNA and RNA-seq collected from the same samples. In the response to the editor's query "Comparison with Other Tools: Discuss the advantages and differences of InterOmics compared to other existing technologies in the multiomic landscape.", the authors respond "due to the low number of samples we have not been able to use other OMICs integrated tools." I do not think this sufficiently addresses the key question, which is no more and no less than "when should a researcher use the framework vs. other tools?" I would argue this is not a new problem. There are other such frameworks: this is the entire purpose of eQTL analysis. But of course eQTL requires very large sample sizes, and applying it to data from six patients would be unreasonable. I think there is an opportunity for the authors to discuss the range of DNA/RNA-seq tools and frameworks in popular use, their assumptions (such as sample size recommendations), and their goals (for instance, some do eQTL analysis, whereas others attempt to refine SNV identification), and explain where InterOmics fits in. The authors are on the right track -- the emphasis should be on relatively small studies, probably on the scale of clinical or pilot investigations, where other tools are not going to be appropriate. But it is necessary to make this comparison and motivation explicit, which means a review of existing literature and methods.

**Reply**

Thank you for this important and constructive comment. We appreciate the opportunity to further clarify the positioning of InterOmics within the landscape of multiomic analytical frameworks.

The primary goal of InterOmics is to enable meaningful integration of DNA and RNA sequencing data in studies with very small sample sizes, such as clinical case series or pilot investigations, where classical approaches like eQTL mapping, which typically require hundreds or thousands of samples, would be statistically infeasible. Traditional frameworks, such as:

- eQTL Analysis Pipelines (e.g., Matrix eQTL, FastQTL) aim to identify associations between genomic variants and gene expression, but they require large cohorts to achieve the statistical power necessary to correct for multiple testing and to account for confounders such as population structure.
- MOFA (Multi-Omics Factor Analysis) and iClusterPlus aim to find shared latent factors that drive variations across multi-omics datasets. They are unsupervised machine learning approaches, excellent for large, heterogeneous cohorts aiming at disease subtype discovery, but they are less suited for small, hypothesis-driven investigations.

In contrast, InterOmics was designed specifically for small-scale studies (e.g., less than 10 subjects), with an emphasis on individual-level variant interpretation rather than on broad population-level inference. The key distinguishing features of InterOmics are:

1. Variant-centric framework: Rather than aiming to discover associations or new sample clusters, InterOmics focuses on classifying variants into biologically meaningful categories (Allele-specific expression, RNA editing, Nonsense-mediated decay, Gain-of-function) per individual.
2. Clinical and translational focus: The structure of InterOmics aims to generate clinically actionable hypotheses at the individual level, for example, inferring whether a variant might be functionally relevant based on its impact on gene expression in the same sample.
3. Multiomic Biological Integration: Because InterOmics does not rely on statistical association models that require correction for multiple testing across a population, it is well-suited for pilot studies, rare disease cohorts, or early-phase precision medicine projects, thus tolerates small sample sizes.

Thus, InterOmics is intended to complement, rather than compete with large-scale methods like eQTL mapping and MOFA. It fills a critical gap where comprehensive genomic-transcriptomic integration is needed, but large datasets are not available.

Following the reviewer’s suggestion, we have expanded the Discussion section to include a comparative overview of current frameworks, their assumptions, and the specific niche addressed by InterOmics.

These paragraphs have been added in the discussion of the manuscript

“Numerous frameworks have been developed for the joint analysis of DNA and RNA sequencing data, each tailored to specific study designs and objectives. Expression Quantitative Trait Loci (eQTL) analysis pipelines (e.g., Matrix eQTL, FastQTL) seek to associate genetic variants with transcript abundance but typically require very large sample sizes (n > 200) to achieve sufficient statistical power and to adjust for multiple testing and population structure confounders. Similarly, unsupervised multi-omics clustering frameworks like MOFA and iClusterPlus are highly effective for large heterogeneous cohorts, enabling the discovery of disease subtypes or novel biological pathways by extracting latent factors across omics layers.

However, these tools are not easily applicable to small-scale, clinical investigations due to their sample size requirements and underlying assumptions. InterOmics was explicitly designed to address this gap, providing a variant-centric framework that enables individual-level genomic-transcriptomic integration. Rather than performing association analyses or unsupervised clustering, InterOmics classifies genetic variants based on their transcriptional consequences—such as allele-specific expression, RNA editing, nonsense-mediated decay, or gain-of-function—within the same subject.

This focus allows InterOmics to be particularly useful for small sample-size studies typical of pilot projects, rare disease cohorts, or early translational research. It tolerates sample sizes that would render statistical association methods underpowered and instead emphasizes biologically interpretable integration designed to inform hypothesis generation and clinical decision-making. Therefore, InterOmics complements existing frameworks, offering a unique solution when the research context involves a limited number of deeply characterized individuals”.

**Comment**

The other concern I have is that, due to patient privacy concerns and the emphasis on the conceptual framework (rather than algorithmic implementation), there are no raw data or code. This makes it much more challenging for somebody to actually implement this framework in their own research. In addition, right now, the logic of some of the steps (e.g. what does it mean to do "gene quantification" with DESeq2?) is still unclear. I think the paper would be far stronger if it were accompanied by a (relatively brief) analogous example workflow applied to a public DNA/RNA-seq dataset on some reasonably well-characterized tissue, which would take the reader through the logic and essential steps of the analysis, going from raw data to processed tables to their joint analysis. Such a tutorial workflow could be done on just one or two patients/donors, and added as a supplement + uploaded to GitHub. But I recognize that what I am asking for is essentially a small new analysis, not necessarily related to the authors' work in HS.

**Reply**

We agree with the Reviewer about the complexity of multiomics analysis. All authors re-read the methods section to clarify complex sentences. In addition, we also create and included in the manuscript a GitHub repository (<https://github.com/ronaldmoura1989/interomics.git>). The analysis starts from genomic and transcriptome integration empowering researchers to implement our multiomic approach.
